# Adrenal Hypoplasia Congenita Presenting as Congenital Adrenal Hyperplasia

**DOI:** 10.1155/2013/393584

**Published:** 2013-02-12

**Authors:** Jennifer L. Flint, Jill D. Jacobson

**Affiliations:** ^1^Department of Pediatrics, Children's Mercy Hospitals and Clinics, University of Missouri-Kansas City School of Medicine, Kansas City, MO 64108, USA; ^2^Division of Endocrinology and Diabetes, Children's Mercy Hospitals and Clinics, University of Missouri-Kansas City School of Medicine, Kansas City, MO 64108, USA

## Abstract

We report on a patient with genetically confirmed adrenal hypoplasia congenita (AHC) whose presentation and laboratory abnormalities were consistent with the more common condition, congenital adrenal hyperplasia (CAH). The patient presented with failure to thrive and salt wasting. General appearance showed marked hyperpigmentation and normal male genitalia. He displayed mildly elevated 17-hydroxyprogesterone and markedly elevated 11-deoxycortisol levels at baseline and with ACTH stimulation testing. Results were consistent with 11**β**-hydroxylase deficiency. He required glucocorticoids and high doses of mineralocorticoids. The marked elevation in 11-deoxycortisol directed our clinical reasoning away from a hypoplastic condition and towards a hyperplasic adrenal condition. Sequencing of the DAX1 gene (named for dosage-sensitive sex reversal (DSS) locus and the AHC locus on the X chromosome) revealed a missense mutation. A review of the literature revealed that elevated 11-deoxycortisol levels have been noted in kindreds with DAX1 mutations, but only when measured very early in life. A mouse model has recently been described that displays elevated 11-deoxycorticosterone levels and evidence for hyperplasia of the zona glomerulosa of the adrenal gland. We conclude that DAX1 testing may be considered in patients with laboratory evidence of 11**β**-hydroxylase deficiency, especially in those with severe salt wasting.

## 1. Introduction

 The X-linked form of adrenal hypoplasia congenita (AHC) is a rare inherited disorder in which the adult cortical zone of the adrenal gland fails to develop [1]. Its cause is an inactivating mutationin the DAX1 gene [2]. The gene is also known as the NROB1 gene (nuclear receptor subfamily 0, group B, member 1). DAX1 is expressed in the adrenal glands, ovaries, testes, and the developing hypothalamus and pituitary. This gene works as a repressor of steroidogenesis factor 1 (SF1), thereby playing a crucial role in suppressing steroidogenesis [3, 4]. Given the role of DAX1 in repressing steroidogenesis, the symptoms in patients with inactivating mutations in DAX1 appear paradoxical. Patients with X-linked adrenal hypoplasia congenita present with signs of combined glucocorticoid and mineralocorticoid deficiency [5]. Its distinction from congenital adrenal hyperplasia is imperative, as the treatments and prognoses differ.

## 2. Case Report

A 21-day-old white male presented to the primary pediatrician for poor feeding, who noted that he had not regained his birth weight. Electrolytes were ordered as part of a failure to thrive workup, which revealed a sodium of 106 mmol/L, a potassium of 7.1 mmol/L, and a glucose of 1.8 mmol/L. After having a 17-OH progesterone level drawn, the infant was transferred to our tertiary referral hospital for electrolyte derangements with the presumptive diagnosis of salt-wasting congenital adrenal hyperplasia.

Birth history was significant for an uneventful pregnancy and delivery. Birth length was 51 cm (65th Percentile). Birth weight was 3.35 kg (37th percentile). Apgar scores were 8 and 9. Hyperpigmentation of the scrotum was noted at birth. Hypoglycemia was noted on the first day of life. He was discharged on the second day of life.

The past medical history was significant for two previous admissions for unconjugated hyperbilirubinemia with a maximum bilirubin of 367 *μ*mol/L.He was treated with phototherapy on both occasions. His parents noted that his skin seemed to be progressively more pigmented over the first three weeks of life. This “bronzing” was attributed to phototherapy.

Physical exam upon arrival revealed normal vital signs and a blood pressure of 68/33 mmHg. His weight was 3.1 kg (7th Percentile). No dysmorphic features were noted. Genital exam revealed normal male genitalia with both testes descended. Phallus was normal in length and caliber with the urethral meatus at the tip. Physical exam was remarkable for marked bronzing of the skin.

Once in the pediatric intensive care unit, the patient was started on fludrocortisone and intravenous fluid support. He underwent a high-dose ACTH stimulation test and then was begun on glucocorticoid treatment at an initial dose of 28 mg/m^2^/day. Severe hyponatremia persisted despite the administration of 400 mcg/day of fludrocortisone in addition to 20 mEq/kg/day of sodium chloride. Diarrhea ensued. Escalating doses of glucocorticoid up to 54 mg/m^2^ were used. By the age of 5 months, he was weaned off of salt supplementation, and hydrocortisone doses were weaned to physiologic levels. Fludrocortisone doses have been gradually reduced.

The11-deoxycortisol values of theACTH stimulation became available early in this hospitalization and were consistent with 11*β*-hydroxylase deficiency with markedly elevated baseline and stimulated levels of 11-deoxycortisol (see Table 1). The 17-hydroxyprogesterone obtained from his primary care physician returned at5.6 pmol/L (normal up to 2.9 pmol/L). As 11-deoxycorticosterone (DOC) and 11-deoxycortisol have been reported to be elevated in 21-hydroxylase deficiency and because salt wasting does not occur in 11-hydroxylase deficiency, 21-hydroxylase deficiency remained the presumptive diagnosis. The following day the baseline and stimulated 17-hydoxy progesterone levels returned to normal levels (4.5 nmol/L), arguing against 21-hydroxylase deficiency. He was evaluated for possible 11*β*-hydroxylase deficiency. A repeat 11-deoxycortisol after 12 days of hydrocortisone treatment returned to 1.49 nmol/L (normal range <.346–4.5).

Genetic testing was sent for CYP11B1 gene. The coding exons and the flanking intronic sequences were PCR amplified and sequenced in forward and reverse directions, using automated fluorescent dideoxy sequencing methods and the mRNA isoform NM_000497 as the reference sequence.

Genetic testing was also performed for the DAX1 (NROB1) gene associated with X-linked congenital adrenal hypoplasia. The coding exons and the flanking intronic sequences were PCR amplified and sequenced in forward and reverse directions, using automated fluorescent dideoxy sequencing methods and the NCBI reference files U31929.Gbk (DAX1, mRNA) and NM_000475.Gbk (NROB1, mRNA) as the reference sequences. This testing was positive for a new missense mutation not previously described. A T to C base change in the second exon was found, which substitutes proline for leucine at codon 447. His mother tested positive as a carrier for this gene. He has two older male siblings who have undergone genetic testing and have been found to be unaffected.

## 3. Discussion

Herein, we describe marked elevations in 11-deoxycortisol (compound S) in a patient with isolated AHC resulting from a missense mutation in the DAX1 gene. A careful review of the literature revealed occasional reports of elevations in 11-deoxycortisol levels in other kindreds with known DAX1 mutations, but only when measured early in life. In one report, elevated 11-deoxycortisol levels were noted in a presymptomatic patient who was the 8-month-old brother of a proband with a known DAX1 mutation [6]. A second report describes three patients with AHC, the youngest of whom displayed elevated 11-deoxycortisol levels when measured at 2 weeks of age [7]. A third report on a large series of patients from several kindreds with DAX1 mutations demonstrated elevated 11-deoxycortisol levels in a patient at one month of age, which spontaneously normalized after six months of life [5].

In the case of kindreds with multiple family members known to carry DAX1 mutations,a complete hormonal diagnostic workup can be anticipated and performed early in life prior to the development of adrenal crises and prior to instituting emergency treatment. In the absence of family histories of DAX1 mutations (and in the absence of other X-linked conditions), several factors may limit the number of patients who would undergo compound S measurement. Patients with isolated AHC will present in severe adrenal crises early in life, at a time when blood volume is extremely limited and emergency hydrocortisone treatment is indicated. Furthermore, neither AHC nor 11*β*-hydroxylase deficiency is at the top of the differential diagnosis in an infant with a severe salt-losing crisis.In textbook cases, 11*β*-hydroxylase deficiency is associated with sodium retention, hypertension, and mineralocorticoid excess, attributed to accumulation of the weak mineralocorticoids, 11-deoxycorticosterone (DOC), rather than with salt loss. Because infants are known to display a relative resistance to mineralocorticoids [8], and because salt loss has been infrequently reported in 11*β*-hydroxylase deficiency, we did consider the possibility of 11*β*-hydroxylase deficiency in our patient [9–11].

The possible association of elevated 11-deoxycortisol levels with inactivating DAX1 mutations becomes more intriguing with the recent generation of the DAX1 knockout mouse model. Mice with inactivating DAX1 mutations display evidence of hyperplasia of the zona glomerulosa, which wanes over time. They exhibit marked elevations in the 11-hydroxycorticosterone (DOC), but only early in life [12]. (Rodents utilize corticosterone rather than cortisol as their major glucocorticoid, presumably resulting from low 17*α*-hydroxylase activity [13]). Over time, DAX1 knockout mice develop adrenal hypoplasia and insufficiency.

If transient marked elevations in 11-hydroxypregnane compounds (11-deoxycortisol and DOC) are indeed a relatively consistent early feature in both mice and humans with inactivating DAX1 mutations, this finding may be clinically relevant. Elevated levels of 11-deoxycortisol may easily confuse clinicians, directing our thoughts away from the possibility of hypoplasia and toward a form of hyperplasia of the adrenal glands, as occurred in our case. On the other hand, if elevations in 11-deoxycortisol could be shown to be an early feature of DAX1 deficiency, they may be useful as early diagnostic tools or possibly as screening tests in AHC.

The hyperplasia of the zona glomerulosa in mice may aid in clarifying a paradox in the DAX1 literature. DAX1 is best known as a repressor to steroidogenesis factor 1 (SF-1), which is an inducer of adrenal and gonadal development and steroidogenesis. If DAX1 represses steroidogenesis, it has seemed paradoxical that inactivating mutations are associated with adrenal hypoplasia. The transient hyperplasia seen in the mouse model of AHC is actually more consistent with expectations in the face of loss of the repressor function. At least one AHC patient has been described who displayed transient glucocorticoid sufficiency, which waned over time [5].

The paradoxical observation of adrenal hypoplasia with inactivation of a steroid repressor has been addressed by the observation that, under certain circumstances, DAX1 can function as an enhancer of steroidogenesis [12]. DAX1 can form coactivator complexes that increase the expression of a subset of steroidogenic genes in the adrenal and gonadal cells [14, 15].

Separate from its role in steroidogenesis, DAX1 appears to be important in differentiation of adrenal progenitor cells. It has been demonstrated that, in the absence of DAX1, the genes involved in steroid hormone production are prematurely hyperactivated.Indeed, knockdown of the DAX1 gene in embryonic stem cells results in spontaneous adrenal differentiation [16, 17]. It is thought that premature activation of DAX1 may impair normal differentiation and zonation of the adrenal gland [15].

A recurring paradox in the congenital adrenal hyperplasia (CAH) literature is the numerous case reports describing “apparent combined” 21-hydroxylase and 11*β*-hydroxylase deficiencies [18–22]. These reports have been enigmatic, given the distant chromosomal locations of the genes encoding the two enzymes. Considering the cortisol synthesis pathway, it is not surprising to see elevations of 17-hydroxyprogesterone in 11*β*-hydroxylase deficiency. More enigmatic have been the reports of elevations of 11-deoxycortisol and DOC in molecularly confirmed 21- hydroxylase deficiency [20]. In the latter scenario, cortisol precursors are accumulating distal to the enzymatic block. A “dysmaturity” of the 11*β*-hydroxylase enzymatic function has been implicated in the elevations in 11-DOC in the presence of 21-hydroxylase deficiency [20]. Other authors have attributed these findings to selective inhibition of 11*β*-hydroxylase by adrenal androgens acting as competitive inhibitors or pseudosubstrates for this enzyme [20, 22]. We speculate that similar mechanisms may be at play, leading to transient elevations in 11-deoxycortisol levels in both DAX1 mutations and in 21-hydroxylase deficiency. Likewise, similar mechanisms may be at play to lead to elevated DOC in mice with DAX1 mutations early in life.

Further studies measuring 11-deoxycortisol and DOC in patients with suspected CAH and AHC prior to the institution of glucocorticoids may help determine how common these elevations are in congenital adrenal disorders.

This case demonstrates that an elevation of 11-deoxycortisol may be a relatively frequent finding in patients with AHC, but perhaps only early in life. This case illustrates a parallelism between human AHC and the mouse model of AHC, which displays transient adrenal hyperfunction which wanes over time. This case also highlights the importance of confirmatory etiologic testing in all cases of unexplained adrenal insufficiency in infancy.

## Figures and Tables

**Table 1:** Adrenal testing results.

	0 minute	Normal (0)	60 minutes	Normal (60)
Cortisol (nmol/L)	56.6*	≥83	60.2*	≥500
17 *α*-(OH) Progesterone (nmol/L)	3.5	.09–2.9	4.5	<8.4
DHEA (nmol/L)	1.7	1.7–26.3	1.8	2.3–50.4
11-Deoxycortisol (nmol/L)	36**	<.346–4.5	37.6**	2.9–11.3
11-Deoxycorticosterone (nmol/L)	.91	.23–1.6	.91	1.2–4.7
Aldosterone (nmol/L)	<.06	NA	NT	NA
Renin (*μ*g/L/h)	189**	2.4–37	NT	NA

*Markedly suppressed.

**Markedly elevated.
